# Modulation of the Endocannabinoid System as a Potential Anticancer Strategy

**DOI:** 10.3389/fphar.2019.00430

**Published:** 2019-05-09

**Authors:** Robert Ramer, Rico Schwarz, Burkhard Hinz

**Affiliations:** Institute of Pharmacology and Toxicology, Rostock University Medical Center, Rostock, Germany

**Keywords:** cancer, endocannabinoids, endocannabinoid-like substances, cannabinoid receptors, fatty acid amide hydrolase, monoacylglycerol lipase

## Abstract

Currently, the involvement of the endocannabinoid system in cancer development and possible options for a cancer-regressive effect of cannabinoids are controversially discussed. In recent decades, a number of preclinical studies have shown that cannabinoids have an anticarcinogenic potential. Therefore, especially against the background of several legal simplifications with regard to the clinical application of cannabinoid-based drugs, an extended basic knowledge about the complex network of the individual components of the endocannabinoid system is required. The canonical endocannabinoid system consists of the endocannabinoids *N*-arachidonoylethanolamine (anandamide) and 2-arachidonoylglycerol as well as the G_i/o_ protein-coupled transmembrane cannabinoid receptors CB_1_ and CB_2_. As a result of extensive studies on the broader effect of these factors, other fatty acid derivatives, transmembrane and intracellular receptors, enzymes and lipid transporters have been identified that contribute to the effect of endocannabinoids when defined in the broad sense as “extended endocannabinoid system.” Among these additional components, the endocannabinoid-degrading enzymes fatty acid amide hydrolase and monoacylglycerol lipase, lipid transport proteins of the fatty acid-binding protein family, additional cannabinoid-activated G protein-coupled receptors such as GPR55, members of the transient receptor family, and peroxisome proliferator-activated receptors were identified as targets for possible strategies to combat cancer progression. Other endocannabinoid-related fatty acids such as 2-arachidonoyl glyceryl ether, *O*-arachidonoylethanolamine, *N*-arachidonoyldopamine and oleic acid amide showed an effect via cannabinoid receptors, while other compounds such as endocannabinoid-like substances exert a permissive action on endocannabinoid effects and act via alternative intracellular target structures. This review gives an overview of the modulation of the extended endocannabinoid system using the example of anticancer cannabinoid effects, which have been described in detail in preclinical studies.

## Introduction

The endocannabinoid system encompasses the two “classical” endocannabinoids, *N*-arachidonoylethanolamine (anandamide, AEA) and 2-arachidonoylglycerol (2-AG), and the cannabinoid receptors CB_1_ and CB_2_. In recent years, further components have expanded this original definition of the endocannabinoid system. These components comprise newly discovered endogenous cannabinoid receptor ligands such as 2-arachidonoyl glyceryl ether (noladin ether, 2-AGE), *O*-arachidonoylethanolamine (virodhamine), *N*-arachidonoyldopamine (NADA), oleic acid amide (oleamide, OA) as well as further receptor targets such as G protein-coupled receptor (GPR) 55 and peroxisome proliferator-activated receptors (PPARs) (Iannotti et al., 2016). Moreover, the cation channel transient receptor potential vanilloid 1 (TRPV1) has been described as an additional receptor, activated by AEA (Zygmunt et al., 1999).

As further parts of the endocannabinoid network, endocannabinoid-synthesizing and -degrading enzymes play a pivotal role in cellular signaling. Accordingly, *N*-acyl-phosphatidylethanolamine-specific phospholipase D (NAPE-PLD), a/b-hydrolase domain-containing protein 4 (ABDH4), glycerophosphodiesterase-1 (GDE1) and tyrosine-protein phosphatase non-receptor type 22 (PTPN22) were identified as AEA-synthesizing enzymes. Diacylglycerol lipase a and -b (DAGL a and -b) were identified as the main 2-AG-producing enzymes. Conversely, AEA and 2-AG were shown to be degraded by fatty acid amide hydrolase (FAAH) with 2-AG being predominantly hydrolyzed by monoacylglycerol lipase (MAGL) (for review, see Di Marzo, 2009; Petrosino and Di Marzo, 2010).

**FIGURE 1:**
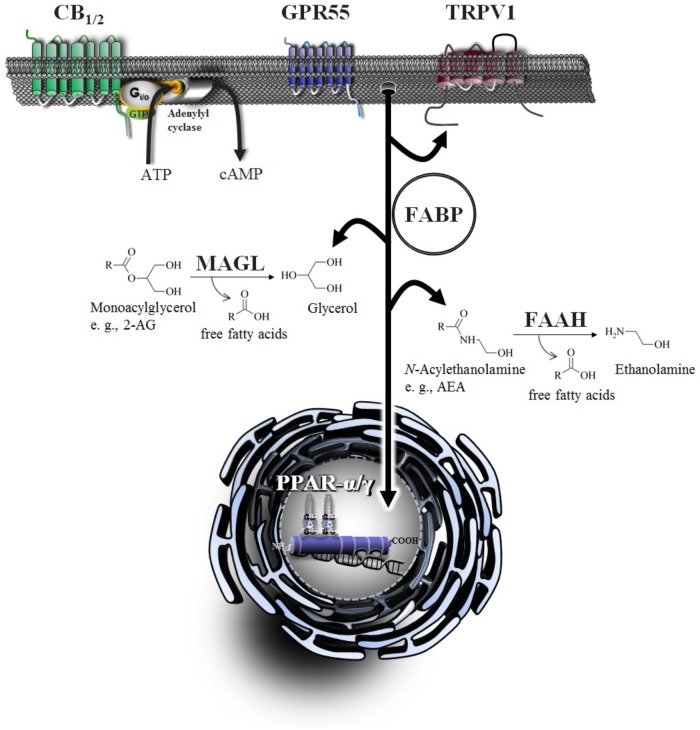
Signaling and degradation of endocannabinoid compounds within the extended endocannabinoid system. CB_1_ and CB_2_, cannabinoid receptors type-1 and -2; FAAH, fatty acid amide hydrolase; FABP, fatty acid binding protein; GPR55, G protein-coupled receptor 55; MAGL, monoacylglycerol lipase; PPAR, peroxisome proliferator-activated receptor; TRPV, transient receptor potential vanilloid type.

In addition to the palliative effects of cannabinoid compounds in cancer treatment, the endocannabinoid system provides several targets for systemic anticancer treatment. Accordingly, preclinical studies suggest cannabinoids inhibit cancer progression via inhibition of cancer cell proliferation, neovascularization, invasion and chemoresistance, as well as induction of apoptosis, autophagy and increase of tumor immune surveillance (for review, see Schwarz et al., 2018). The following chapters focus on the different levels of anticancer effects of cannabinoids and the role of components of the endocannabinoid system within this process. An overview of important elements of the endocannabinoid system is provided in Figure 1.

## Regulation of Cannabinoid Receptors in Malignant Tissue

The scientific basis of the endocannabinoid system was established with the discovery and cloning of a central seven-transmembrane G protein-coupled cannabinoid receptor, referred to as CB_1_ (Matsuda et al., 1990). Subsequently, the discovery of the peripheral CB_2_ receptor on spleen and blood cells followed in 1993 (Munro et al., 1993). Initial analysis of intracellular pathway coupling revealed both receptors to act via pertussis toxin-sensitive G_i/o_ protein interaction, resulting in inhibition of adenylyl cyclase activity and thus intracellular reduction of cAMP levels (Das et al., 1995; Slipetz et al., 1995).

Concerning the regulation of cannabinoid receptors in malignant tissue, a number of investigations provided evidence for an upregulation of cannabinoid receptors in cancer tissue (for review, see Ramer and Hinz, 2016). In this context, a correlation between high CB_1_ receptor expression and poor prognosis for patients with pancreatic (Michalski et al., 2008), prostate (Chung et al., 2009), ovarian (Messalli et al., 2014), colorectal cancers (Jung et al., 2013) and renal cell carcinoma (Wang J. et al., 2016) was shown. However, concerning renal carcinomas, CB_1_ receptor expression was found to appear downregulated in clear cell renal cell carcinoma (Larrinaga et al., 2010) as well as in chromophobe renal cell carcinoma and renal oncocytoma (Larrinaga et al., 2013).

In terms of higher CB_2_ receptor expression, a link to poor prognosis for patients with HER2-positive breast cancer (Pérez-Gómez et al., 2015), as well as for head and neck squamous cell carcinoma (HNSCC) (Klein Nulent et al., 2013) was demonstrated. Furthermore, immunohistochemical analyses of biopsies from human astrocytoma revealed detectable cannabinoid receptors (CB_1_ and/or CB_2_) in 70% of all cases (Sánchez et al., 2001). Notably, analyses of the CB_2_ receptors exhibited a significant number of biopsies with high/very high expression of CB_2_ receptors in grade IV astrocytoma versus grade I–III astrocytoma suggesting the CB_2_ receptor expression to correlate with malignancy. Another comprehensive study that addressed the regulation of cannabinoid receptors in hepatocellular carcinoma found high expression of both CB_1_ and CB_2_ receptors in 45% and 52% of all cancer tissue samples by immunohistochemical analyses, respectively (Xu et al., 2006). Here, the authors showed high CB_1_ receptor expression in 87% of well-differentiated cancers and high CB_2_ receptor expression in 100% of well-differentiated cancers versus low expression of the CB_1_ as well as the CB_2_ receptor in 73% of poorly differentiated cancers, respectively. These findings correspond to the mRNA levels assessed by *in situ* hybridization, suggesting that expression of cannabinoid receptors decreased according to cell differentiation. Disease-free survival was significantly improved for patients with hepatocellular carcinoma that exhibited high CB_1_ and CB_2_ expression as compared to those with low cannabinoid receptor expressions. Concerning the regulation of cannabinoid receptors in glioma, another study that assessed cannabinoid receptor expression in malignant tissue was able to demonstrate detectable CB_2_ in three out of six low-grade gliomas but in all high-grade gliomas (Calatozzolo et al., 2007). In the latter study, two out of six low-grade gliomas and five out of eight high-grade gliomas were positive to CB_1_. Furthermore, a recent study found CB_2_ to be upregulated in renal cell carcinoma. Here a tendency of higher CB_2_ expression was associated with poor clinical outcome (Wang et al., 2018).

An important contribution to CB_1_ receptor regulation in cancer tissue may apparently be epigenetic regulations. A recent investigation on colon cancer progression found CNR1 methylation increased at CpG islands surrounding the promoter region, whereas it was decreased in the body of the gene in tumor samples. The authors further found CB_1_ mRNA to appear upregulated in non-tumor tissue and downregulated in tumor tissue (Hasenoehrl et al., 2018). Another study confirmed epigenetic hypermethylation of the CNR1 promoter that results in reduced transcription (Wang D. et al., 2008). Analyses of patients’ specimens here revealed CB_1_ mRNA and protein to appear drastically reduced in cancer tissue as compared with normal mucosa.

## Endocannabinoids and Endocannabinoid-Like Substances

The first endogenously synthesized compounds proven to act at cannabinoid receptors were AEA (Devane et al., 1992) and 2-AG (Mechoulam et al., 1995). AEA was identified as a partial agonist at the CB_1_ with a receptor affinity comparable to that of the phytocannabinoid Δ^9^-THC (Devane et al., 1992; Mackie et al., 1993; Sugiura et al., 1999; Di Marzo and De Petrocellis, 2012), while being almost inactive at the CB_2_ receptor (Gonsiorek et al., 2000; Sugiura et al., 2000; Di Marzo and De Petrocellis, 2012). 2-AG, however, was shown to exert full agonist properties with a moderate affinity at both cannabinoid receptors (Sugiura et al., 1999, 2000; Gonsiorek et al., 2000; Savinainen et al., 2001).

As has been proven for the cannabinoid receptors, the levels of endocannabinoids in malignant tissue were likewise shown to be elevated. Accordingly, concentrations of AEA and 2-AG were found to be increased in adenomatous polyps and in colorectal carcinomas when compared with healthy neighboring tissue (Ligresti et al., 2003). In agreement with this finding, increases of endocannabinoid concentrations were detected in pituitary adenomas (Pagotto et al., 2001), in prostate (Schmid et al., 2002; Nithipatikom et al., 2004) and colorectal cancers (Chen et al., 2015), as well as in meningiomas and glioblastomas (Petersen et al., 2005).

With respect to the functional implication of endocannabinoids in tumor progression, as early as two decades ago AEA was shown to confer a concentration-dependent inhibitory effect (maximal inhibition at 10 μM) on the proliferation of nerve growth factor-activated breast cancer cells via activation of CB_1_ receptors and downstream inhibition of endogenous prolactin action (De Petrocellis et al., 1998). Another early investigation on that topic confirmed the involvement of the CB_1_ receptor in the AEA-induced inhibition of nerve growth factor-activated breast and prolactin-activated prostate cancer cell proliferation (Melck et al., 2000). Later investigations were able to further verify this anticancer effect. Accordingly, AEA in the range of 0.01 to 10 μM was found to elicit an antiproliferative action on glioma cells via both cannabinoid receptors and TRPV1 by enhancing downstream oxidative stress and calpain activation (Jacobsson et al., 2001). Inhibition of colon carcinoma cell proliferation by AEA at 1 μM was reversed by an antagonist to CB_1_, but not CB_2_ (Ligresti et al., 2003). Using epidermal growth factor (EGF)-activated prostate cancer cells, another study was able to demonstrate AEA to inhibit proliferation by downregulation of EGF receptor expression via upstream activation of the CB_1_ receptor (Mimeault et al., 2003). Besides this antiproliferative action, AEA was further shown to inhibit lung cancer cell invasion and metastasis via upregulation of tissue inhibitor of matrix metalloproteinases-1 (TIMP-1) (Winkler et al., 2016) and to induce apoptosis in human gastric adenocarcinoma (Ortega et al., 2016). Moreover, AEA was demonstrated to induce a cyclooxygenase-2 (COX-2)-dependent cell death in apoptosis-resistant colon cancer cells at a concentration of 25 μM devoid of CB_1_ and CB_2_ receptor activation (Patsos et al., 2010). In contrast to these findings, another investigation could not confirm a contribution of COX-2 to the AEA-induced apoptosis of HNSCC cell lines (Park et al., 2015). In this study, a high concentration of AEA (20 μM) was shown to induce apoptosis via enhanced oxidative stress bypassing cannabinoid-activated receptors. In an investigation that addressed the cytotoxic effect of AEA on malignant melanoma cells, a decrease of AEA-induced cytotoxicity was observed in the presence of the selective COX-2 inhibitor rofecoxib and the lipoxygenase (LOX) inhibitor caffeic acid (Adinolfi et al., 2013). In addition, the authors were able to demonstrate a rightward shift of the concentration–response curve of AEA in the presence of the CB_1_ receptor antagonist AM-251 and the TRPV1 antagonist capsazepine. Accordingly, the AEA-induced loss of viability appeared with an IC_50_ level of 5.8 ± 0.7 μM that was shifted up to 10.9 ± 0.6 μM and 8.2 ± 0.4 μM in the presence of AM-251 and capsazepine, respectively, indicating at least a partial contribution of the CB_1_ receptor to the toxic effect of AEA on melanoma cells. Concerning the anticarcinogenic effect on glioblastoma cells, another investigation found AEA to inhibit proliferation, to accumulate cells in the G_0_/G_1_ phase, to reduce percentage of cells in the G_2_/M phase, to inhibit cellular migration and to induce apoptosis within a concentration range from 1 to 10 μM in a concentration dependent manner (Ma et al., 2016). Concerning the involvement of cannabinoid-activated receptors, there is a tendency for receptor-independent effects of AEA when tested at concentrations >10 μM.

Furthermore, several AEA derivates have been demonstrated to likewise exert anticancer properties. Accordingly, arachidonoyl-2′-chloroethylamide (ACEA) was found to cause antiproliferative effects on colorectal carcinoma cells at concentrations ranging from 0.01 to 1 μM (Ligresti et al., 2003). Two other AEA derivates with proven anticancer properties are R(+)-methanandamide and 2-methyl-arachidonyl-2′-fluoro-ethylamide (Met-F-AEA). R(+)-methanandamide (10 μM) was shown to elicit apoptosis via a mechanism bypassing cannabinoid and TRPV1 activation (Hinz et al., 2004; Eichele et al., 2006, 2009) in cervical and lung cancer as well as glioma cells. However, an involvement of CB_1_ and CB_2_ receptors on mantle cell lymphoma apoptosis (Gustafsson et al., 2006) and of CB_1_ on apoptosis of prostate cancer cells (Orellana-Serradell et al., 2015) was demonstrated. Induction of apoptosis by R(+)-methanandamide at a concentration of 5 μM was further confirmed in gastric cancer cells (Ortega et al., 2016). In addition, R(+)-methanandamide caused a cannabinoid receptor- and TRPV1-dependent inhibition of cervical and lung cancer cell invasion (Ramer and Hinz, 2008) and an enhancement of lung cancer cell killing by lymphokine-activated killer cells in a coculture system via upregulation of the intercellular adhesion molecule 1 (ICAM-1), the counterreceptor of the lymphocyte-function-associated antigen 1 (LFA-1) on the surface of killer cells (Haustein et al., 2014). A contribution of the CB_1_ receptor to the cancer growth-inhibitory action of Met-F-AEA (10 μM) was demonstrated using ras oncogene-dependent tumor cells (Bifulco et al., 2001) and colorectal cancer cells (Proto et al., 2012). Moreover, Met-F-AEA caused inhibition of metastasis of Lewis lung carcinoma (Portella et al., 2003) and breast cancer (Grimaldi et al., 2006). In addition, a cell cycle arrest by suppression of Cdk2 activity could be proven for Met-F-AEA in breast cancer cells (Laezza et al., 2006). The same investigators later described 10 μM Met-F-AEA to reduce the expression of β-catenin protein and its accumulation in the nuclei of breast cancer cells via CB_1_ receptor activation associated with downregulation of c-Myc, matrix metalloproteinase-2 (MMP-2) and cyclin D1 (Laezza et al., 2012). The latter study further found Met-F-AEA to cause downregulation of mesenchymal markers such as vimentin, fibronectin and N-cadherin by induction of E-cadherin and upregulation of the markers indicating epithelial-to-mesenchymal transition such as Snail, Slug, and Twist in breast cancer cells.

Similar to AEA, 2-AG was likewise demonstrated to exert considerable anticancer properties. Accordingly, activation of the CB_1_ receptor could be proven to contribute to the antiproliferative action of 2-AG on nerve growth factor-stimulated breast cancer cells (Melck et al., 2000) and to its proapoptotic action on prostate cancer cells (Orellana-Serradell et al., 2015) when using 2-AG concentrations in the range of 0.5–5 μM and 5 μM, respectively.

Other cancer entities sensitive toward 2-AG are colorectal carcinoma (Ligresti et al., 2003) and glioma cells (Jacobsson et al., 2001). A recent study was able to demonstrate 2-AG to inhibit lung cancer cell invasion in a concentration dependent manner (0.01–10 μM) (Winkler et al., 2016) and to further decrease metastasis *in vivo*. Finally, 2-AG was proven to inhibit proliferation (Nithipatikom et al., 2011) and invasion (Nithipatikom et al., 2004) of prostate carcinoma cells.

Concerning the molecular effects of endogenously synthesized cannabinoid compounds beyond the two classical endocannabinoids AEA and 2-AG, ambivalent data have been published so far. An early study revealed NADA to exert preferential 40-fold higher affinity to the CB_1_ (*K*_i_ value of 250 nM) compared to CB_2_ receptors (Bisogno et al., 2000). However, another study could not find NADA to act as CB_1_ agonist but to enhance mobilization of calcium via *G*_q_ protein-dependent processes (Redmond et al., 2016). Another investigation was able to provide evidence for NADA to elicit intracellular calcium increase via TRPV1 (Huang et al., 2002). As further mode of action, NADA may also inhibit FAAH activity with an IC_50_ value of 23 μM, thereby causing increased levels of classical endocannabinoids (Bisogno et al., 2000). Noladin ether was shown to act as an agonist at the CB_1_ and CB_2_ receptors and as partial TRPV1 agonist (Hanus et al., 2001; Duncan et al., 2004). In contrast, virodhamine was found to function as a CB_1_ receptor antagonist and a CB_2_ receptor agonist (Porter et al., 2002). As has been demonstrated for NADA, virodhamine was likewise described as a FAAH inhibitor (IC_50_ value of 13.8 μM) (Steffens et al., 2005) and furthermore as an inhibitor of MAGL (Brantl et al., 2014). Other substances shown to act as cannabinoid receptor agonists are *N*-docosahexaenoylethanolamine (DHEA) and *N*-eicosapentaenoylethanolamine (EPEA) (Brown et al., 2010). A possible common mode of action discussed for the endocannabinoids AEA and 2-AG, as well as for NADA, noladin ether and virodhamine, may lie in their agonistic action at GPR55 (Ryberg et al., 2007; Akimov et al., 2017). EC_50_ values of the endocannabinoids were found in a low-nanomolar range (3 nM for 2-AGE, up to 18 nM for AEA) at the GPR55, which even appeared lower than those evaluated for the CB receptors (lowest values measured for AEA were 31 nM at the CB_1_ and 27 nM at CB_2_) (Ryberg et al., 2007).

*N*-Palmitoylethanolamine (PEA), *N*-oleoylethanolamine (OEA), and *N*-stearoylethanolamine (SEA) are referred to as cannabinoid-like substances that do not elicit cannabinoid receptor activation (Bisogno et al., 1998; Maccarrone et al., 2002). Among these, PEA was demonstrated to permissively enhance AEA effects by downregulation of FAAH expression and activity and via positive allosteric modulation of TRPV1, which was termed the “entourage effect” (Di Marzo et al., 2001; De Petrocellis et al., 2002). Currently, the available data concerning the regulation of endocannabinoid-like substances in cancer tissue is limited. One study was able to demonstrate lower levels of OEA and PEA associated with a downregulation of AEA but not with 2-AG in human meningiomas and gliomas (Maccarrone et al., 2001). Another investigation demonstrated higher plasma concentrations of circulating OEA to correlate with higher numbers of metastases in several cancer entities (Sailler et al., 2014). An overview on the regulation of endocannabinoids and endocannabinoid-like substances in cancer tissue is provided in Table 1.

**Table 1:** Regulation of endocannabinoids and endocannabinoid-like substances in cancer tissue.

Tumor	References	Endocannabinoid	Regulation
Colorectal carcinoma	Ligresti et al., 2003	2-AG	↑
		AEA	
	Chen et al., 2015	AEA	↑
		2-AG	↔
Diffuse large B-cell lymphoma	Zhang et al., 2016	2-AG ^a^	↑
		2-OG ^a^	
Endometrial carcinoma	Guida et al., 2010	2-AG	↑
		AEA	↔
		PEA	
Glioma	Maccarrone et al., 2001	AEA	↓
		OEA	
		PEA	
		SEA	
		2-AG	↔
	Petersen et al., 2005	AEA	↑
		2-AG	↔
	Wu et al., 2012	AEA ^b^	↓
		2-AG ^b^	↑
		OEA	↔
Hepatocellular carcinomas	Mukhopadhyay et al., 2015	AEA	↑
Meningioma	Maccarrone et al., 2001	AEA	↓
		OEA	
		PEA	
		SEA	
		2-AG	↔
	Petersen et al., 2005	AEA	↔
		2-AG	↑
Pituitary adenomas	Pagotto et al., 2001	AEA	↑
		2-AG	
Various cancers^d^	Sailler et al., 2014	AEA ^c^	↓
		2-AG^c^	↑
		PEA ^c^	↔
		OEA ^c^	

*↔, not regulated; ↑, upregulated; ↓, downregulated; ^*a*^, measured in patients’ serum; ^b^, associated with advanced disease stages; ^c^, measured in patients’ plasma; ^d^, endocannabinoids measured in plasma from breast, prostate, lung, pancreatic, skin, hematological, gynecological, and other unnamed cancers; 2-AG, 2-arachidonoylglycerol; 2-OG, 2-oleoylglycerol; AEA, N-arachidonoylethanolamine (anandamide); OEA, N-oleoylethanolamine; PEA, N-palmitoylethanolamine; SEA, N-stearoylethanolamine. If no arrow is indicated, the regulation of the endocannabinoid above applies.*

The data concerning effects of cannabinoid agonists such as NADA, noladin ether, and virodhamine or cannabinoid-like substances on tumor progression are currently still limited. An early investigation found NADA to elicit inhibition of breast cancer cell proliferation *in vitro* (IC_50_ value of 0.25 μM) and *in vivo* (Bisogno et al., 2000). Furthermore, a recent study demonstrated antiproliferative and cell death-inducing properties for NADA in human osteosarcoma, neuroblastoma, breast adenocarcinoma, lymphoma, and leukemia cell lines when tested at micromolar concentrations (Akimov et al., 2015). Concerning the mode of action, NADA (1 μM) has been found to confer antiproliferative effects on colorectal carcinoma cells via activation of CB_1_ (Ligresti et al., 2003). In terms of pheochromocytoma cells, an antagonist to GPR55 was able to counteract NADA-induced cell death (Akimov et al., 2017), whereas cell death of neuroblastoma cells by NADA was mediated by TRPV1 activation (Davies et al., 2010). Noladin ether was shown to confer inhibition of prostate cancer cell proliferation (1–50 μM) via nuclear factor (NF)-κB/cyclin D- and cyclin E-dependent pathways (Nithipatikom et al., 2011). The anti-invasive action of noladin ether was mediated by downregulation of protein kinase A activity (Nithipatikom et al., 2004). In contrast, nothing is yet known about the anticarcinogenic effects of virodhamine.

Concerning the effects of fatty acid ethanolamides belonging to the group of endocannabinoid-like substances, OEA and PEA were found to elicit decreased viability of neuroblastoma cells at a concentration of 10 μM (Hamtiaux et al., 2011) and to inhibit invasion and metastasis of lung cancer cells (Winkler et al., 2016). In the case of OEA, the latter study reported that the anti-invasive action was reversed by knockdown of TIMP-1. PEA was shown to inhibit colon carcinoma cell proliferation by inhibition of the Akt/mechanistic target of rapamycin (mTOR) pathway that involves PPARα (Sarnelli et al., 2016), to enhance inhibition of proliferation by AEA by virtue of its action as positive allosteric modulator of TRPV1 (De Petrocellis et al., 2002) and to permissively enhance AEA effects by downregulation of FAAH expression and activity (Di Marzo et al., 2001). A similar entourage effect was reported for SEA (Maccarrone et al., 2002), which has also been found to elicit proapoptotic effects in rat glioma cells by increasing intracellular calcium levels and mitochondrial uncoupling (Maccarrone et al., 2002). Conversely, a recent study reported enhanced migration of melanoma cancer cells as result of treatment with OEA (Sailler et al., 2014). Antiproliferative properties were observed in prostate cancer cells treated with DHEA and EPEA (Brown et al., 2010). In this context, DHEA and EPEA-induced toxicity was found to be associated with increased autophagy in breast cancer cells (Rovito et al., 2013). In addition, a contribution of the LOX pathway was demonstrated for the antiproliferative action of DHEA and *N*-arachidonoyl-L-alanine (NALA) at concentrations of 20 μM each on HNSCC cells (Park et al., 2016). Accordingly, the antiproliferative action of DHEA and NALA was not reversed by the CB_1_ receptor antagonist/inverse agonist AM-251 and the TRPV1 antagonist CAY10448, but by the inhibitors of 5-lipoxygenase (5-LO), AA861, zileuton, ebselen as well as by knockdown of 5-LO using an siRNA approach. As a mechanistic basis of this effect the authors assumed a decrease of the phosphorylated form of Akt by reactive oxygen species generated upon catabolism of DHEA and NALA via 5-LO.

## TRP Channels

Among the transient receptor potential (TRP) family, TRPV1 emerged as an additional “ionotropic cannabinoid receptor” (Di Marzo et al., 2002) that was found to be activated by AEA (Zygmunt et al., 1999) and the non-psychoactive phytocannabinoid cannabidiol (CBD) (Bisogno et al., 2001; Ligresti et al., 2006). Further TRP channels modulated by cannabinoid compounds are TRPV2, which was reported to be activated by CBD (Qin et al., 2008; Nabissi et al., 2013) and Δ^9^-THC (De Petrocellis et al., 2011) as well as the CBD- and tetrahydrocannabivarin-triggered TRPV3 (De Petrocellis et al., 2012). TRPV3 and TRPV4 were further found to be desensitized by cannabigerovarin and cannabigerolic acid (De Petrocellis et al., 2008). Cannabigerol, CBD, and cannabinol were additionally identified as activators of TRP channels of the ankyrin type-1 (TRPA1) and the melastatin type-8 (TRPM8), with the latter receptor also being activated by Δ^9^-THC acid (De Petrocellis et al., 2008).

Among the large number of members of the TRP family, the contribution of TRPV1 to the anticancerogenic effects of cannabinoids is the most comprehensively investigated issue. Accordingly, TRPV1 was shown to be upregulated in malignant prostate cancer tissue (Czifra et al., 2009). Anticancer effects of AEA were described to be mediated via TRPV1 in a number of early studies. As such, AEA was found to induce apoptosis in neuroblastoma, lymphoma (Maccarrone et al., 2000) and cervical cancer cells (Contassot et al., 2004) and to inhibit glioma cell proliferation (Jacobsson et al., 2001) via TRPV1 activation. Inhibition of breast cancer cell proliferation by CBD was further found to be partially mediated via TRPV1 (Ligresti et al., 2006). A causal contribution of TRPV1 to the anti-invasive impact of R(+)-methanandamide on cervical and lung cancer cells was shown to be mediated by upregulation of TIMP-1 (Ramer and Hinz, 2008). A similar anti-invasive TRPV1-dependent impact on lung cancer cells could be proven for FAAH siRNA and the FAAH inhibitors URB597 and *N*-arachidonoylserotonin (AA-5HT) (Winkler et al., 2016), as well as for CBD (Ramer et al., 2012). CBD was further demonstrated to reduce cancerogenesis in an azoxymethane-induced colon cancer model. Here, accompanying *in vitro* experiments showed an involvement of TRPV1 in the antiproliferative action of CBD on colon cancer cells (Aviello et al., 2012). In a recent investigation TRPV1 was finally shown to contribute to the toxicity of AEA and CBD on endometrial adenocarcinoma cells (Fonseca et al., 2018).

According to a recent investigation, TRPM8 inhibition by cannabigerol may represent another mechanism for inhibition of colon carcinogenesis (Borrelli et al., 2014). Furthermore, recent studies suggest TRPV2 to act as key regulator of glioma cell autophagy induced upon CBD treatment (Nabissi et al., 2015) and as mediator of a cannabinoid-induced sensitivity of cancer cells toward chemotherapeutics (Nabissi et al., 2013; Morelli et al., 2014).

## G Protein-Coupled Receptors

Several investigations of recent years have described a number of G protein-coupled receptors (GPRs) activated or inhibited by cannabinoid treatment.

Accordingly, GPR55 was found to be activated by the synthetic regioisomer of CBD, abnormal-CBD (abn-CBD), the specific GPR55 agonist structurally related to cannabinoids, O-1602, as well as by R(+)-methanandamide, Δ^9^-THC and the specific CB_2_ receptor agonist JWH-015 (Johns et al., 2007; Lauckner et al., 2008). In agreement with these findings, AEA and virodhamine were described as cannabinoid compounds acting as agonists at GPR55, whereas CBD was revealed as a GPR55 antagonist (Ryberg et al., 2007). A further receptor target of cannabinoid action among the GPRs may be GPR18, which has been shown to be activated by *N*-arachidonoylglycine (NAGly), O-1602, abn-CBD, Δ^9^-THC and AEA (McHugh et al., 2012). However, several studies did not confirm these results concerning the activity of NAGly, AEA and abn-CBD at GPR18 (Yin et al., 2009; Lu et al., 2013; Finlay et al., 2016). Thus, the definition of GPR18 as a cannabinoid-activated receptor has to be evaluated critically. With respect to other GPRs as members of the endocannabinoid system in an extended definition, OEA and PEA were found to modulate GPR119 (Overton et al., 2006), NAGly was revealed to exert binding to GPR92 (Oh et al., 2008) and finally CBD was found to act as inverse agonist at GPR12 (Brown et al., 2017).

Regarding a possible role in cancer regression by cannabinoid action, GPR55 emerged as a promising target. Notably, GPR55 has been found to be a receptor conferring cancerogenesis when activated with lysophosphatidylinositol (LPI) (Henstridge et al., 2011; Ross, 2011). In agreement with this notion, high GPR55 mRNA expression in tumor tissue of patients with colorectal carcinomas was described to be significantly associated with reduced relapse-free survival (Hasenoehrl et al., 2018). Accordingly, GPR55 mRNA expression levels revealed a downregulation that was associated with increasing TNM stages. The latter investigation further found reduced colorectal carcinogenesis in GPR55 knockout mice. In line with this, several cannabinoids were demonstrated to counteract GPR55 action. Accordingly, CBD acting as GPR55 antagonist was shown to inhibit colon cancerogenesis (Kargl et al., 2016; Hasenoehrl et al., 2018). Furthermore, a crosstalk between CB_2_ receptors and GPR55 was identified as a determinant of cancer progression (Moreno et al., 2014).

## Members of the Ppar Family

In the context of direct modulations of PPARs, the endocannabinoid-like substances EPEA, DHEA, SEA, OEA, and PEA are currently discussed as PPARα agonists (Fu et al., 2003; Lo Verme et al., 2005; Artmann et al., 2008; Tellez et al., 2013). Regarding the effect of Δ^9^-THC on PPARα, one study did not find an activation of PPARα (Sun et al., 2007), whereas another reported Δ^9^-THC-induced transcriptional activity of PPARα in breast cancer cells (Takeda et al., 2014). In line with this, cannabinoids such as WIN 55,212-2, AEA, OEA, virodhamine and noladin ether were revealed as PPARα agonists in reporter gene assays (Sun et al., 2007). As a further cannabinoid target among the PPARs, PPARγ was shown to be activated by Δ^9^-THC (O’Sullivan et al., 2005, 2006), CBD (O’Sullivan et al., 2009) and AEA (Bouaboula et al., 2005).

Several lines of evidence suggest a possible contribution of PPARs to the anticarcinogenic effects of cannabinoids. Accordingly, Δ^9^-THC was found to exert antiproliferative effects on hepatocellular carcinoma cells *in vitro* and *in vivo* via PPARγ (Vara et al., 2013). Similar results were reported for WIN 55,212-2 in hepatocarcinoma cells (Giuliano et al., 2009; Pellerito et al., 2010; Hong et al., 2013). Another study revealed an indirect action of cannabinoids on PPARγ activation. Thus, CBD and R(+)-methanandamide were found to confer PPARγ activation via upregulation of COX-2 expression and subsequent release of prostaglandins, acting as PPARγ ligands (Eichele et al., 2009; Ramer et al., 2013).

## Other Receptor Targets

Accumulating evidence suggests glycine receptors as targets of phytocannabinoid action in context with their anti-inflammatory and antinociceptive properties. Accordingly, Δ^9^-THC and AEA were described to potentiate glycine currents in native neurons, hippocampus, amygdala, and spinal cord (Hejazi et al., 2006). A recent publication further unraveled the voltage-dependent anion channel 1 (VDAC1) as a mitochondrial receptor target of cannabinoids. Thus, microglial cell death was reported to occur through the inhibition of VDAC1 conductance by CBD (Rimmerman et al., 2013). The authors hypothesized that the inhibition of this channel may also be responsible for the anticancer properties of CBD.

## Biosynthesizing Enzymes

The biosynthesis of AEA and other *N*-ethanolamines is based on the transacylation-phosphodiesterase pathway (Schmid, 2000; Wang and Ueda, 2009), which consists of a first step, *N*-acylation of phosphatidylethanolamine by *N*-acyltransferases (Jin et al., 2007; Wang and Ueda, 2009). Subsequently a second step reaction of *N*-acyl-phosphatidylethanolamine-selective phospholipase D (NAPE-PLD) generates AEA. Alternative sequential steps include the action of ABHD4 followed by turnover of the intermediate glycero-phosphatidyl-AEA via glycerophosphodiesterase 1. Another alternative pathway includes conversion of phosphatidylethanolamine via soluble phospholipase A_2_ (sPLA_2_) and subsequent turnover by lysophospholipase D (lyso-PLD). Finally, a third alternative metabolic pathway runs via phospholipase C (PLC) and downstream phosphatases such as PTPN22 and phosphatidylinositol 3,4,5-trisphosphate 5-phosphatase 1 (SHIP1) (Liu J. et al., 2008).

The biosynthesis of 2-AG is based on cleavage of membrane phospholipids via phospholipase C and by diacylglycerol (DAG) turnover via DAG lipase a and -b (Prescott and Majerus, 1983; Stella et al., 1997). Alternative pathways include combined action of phospholipase A_1_ (PLA_1_) and lyso-PLC (Sugiura et al., 1995) or dephosphorylation of arachidonic acid-containing lysophosphatidic acid (Nakane et al., 2002).

Concerning the regulation of endocannabinoid-biosynthesizing enzymes in cancer tissue, currently available data are rare. One investigation reported NAPE-PLD to appear to be downregulated in expression and activity level in glioma tissue, whereas the expression of DAGL remained unchanged (Wu et al., 2012). Notably, this disequilibrium, combined with a downregulation of both degrading enzymes, FAAH and MAGL, resulted in decreased AEA and increased 2-AG concentrations. In another study, however, NAPE-PLD mRNA was found to be upregulated in colorectal cancer tissue (Chen et al., 2015).

## Degradation Enzymes

The main enzymes for endocannabinoid degradation are FAAH and MAGL. FAAH was first defined as the catabolic enzyme for AEA (Deutsch and Chin, 1993) and 2-AG (Di Marzo et al., 1998; Goparaju et al., 1999) and later also for the turnover of other fatty acid derivates such as the endocannabinoid-like substances OA (Cravatt et al., 1996), OEA and PEA (Desarnaud et al., 1995; Saghatelian et al., 2004). The major enzyme of 2-AG hydrolysis is MAGL (Blankman et al., 2007), although other enzymes such as ABHD6 and ABHD12 have also been demonstrated to hydrolyze 2-AG (Blankman et al., 2007). Furthermore, AEA and 2-AG are substrates for oxidation by COX-2, which subsequently results in prostaglandin ethanolamides and prostaglandin glycerol esters (Yu et al., 1997; Kalgutkar et al., 1999; Kozak et al., 2000). Another enzyme degrading AEA, OEA, and PEA is the lysosomal hydrolase *N*-acylethanolamine-hydrolyzing acid amidase (NAAA) (Ueda et al., 1999; Sun et al., 2005). In particular, it has been suggested that NAAA plays a role in prostate carcinoma proliferation, as high expression in prostate carcinoma cells has been demonstrated (Wang J. et al., 2008). In agreement with this notion, one study found *N*-cyclohexanecarbonylpentadecylamine, an inhibitor of NAAA, to elicit decreased viability of neuroblastoma cells (Hamtiaux et al., 2011). A recent study aiming at the design of new NAAA inhibitors reported these NAAA inhibitors to induce bladder cancer cell death and reduce cell migration (Vago et al., 2017).

A number of studies suggest the main enzymes for endocannabinoid turnover, FAAH and MAGL, to appear upregulated in malignant tissue. Accordingly, higher expression levels of FAAH have been found in prostate cancer tissue (Endsley et al., 2008). In addition, a correlation between high FAAH expression and disease severity has been identified (Thors et al., 2010). Increases in MAGL expression were observed in ovarian tumors, in colorectal cancer tissues (Nomura et al., 2010; Ye et al., 2011; Pagano et al., 2017) and in ductal breast tumors compared to less malignant breast tumors (Gjerstorff et al., 2006). Such association was further confirmed for colon tumor cases, where the prognosis for patients with high MAGL expression was markedly poorer than that for those with low MAGL expression (Zhu et al., 2016). In contrast to these findings, high levels of FAAH and MAGL correlate with a positive survival prognosis for patients with pancreatic ductal adenocarcinomas (Michalski et al., 2008). In terms of endometrial carcinoma, another study presented downregulation of MAGL in cancer versus healthy tissue (Guida et al., 2010) which was later confirmed for colorectal, lung, breast, stomach and ovarian cancers (Sun et al., 2013). Notably, these findings support the notion of the endocannabinoid-degrading enzymes being tumor suppressors rather than tumor promoters.

During the last two decades endocannabinoid-degrading enzymes have attracted considerable interest as probable targets of an innovative anticancer treatment. Thus, inhibitors of FAAH such as AA-5HT, which cause reduction of AEA turnover and prolonged action of AEA at cannabinoid receptors, were found to be potent inhibitors of glioma (Jacobsson et al., 2001), colorectal (Ligresti et al., 2003) and thyroid cancer cell proliferation (Bifulco et al., 2004). AA-5HT was further proven to reduce aberrant crypt foci in a murine colon carcinogenesis model (Izzo et al., 2008). URB597, another FAAH inhibitor, was shown to reduce proliferation of neuroblastoma cells when combined with the FAAH substrate AEA (Hamtiaux et al., 2011). Similar results were obtained from experiments in which URB597 was combined with Met-F-AEA on colorectal (Proto et al., 2012) and lung cancer cells (Hamtiaux et al., 2012), with the latter study further demonstrating an antiproliferative impact of combined treatment with URB597 and PEA on melanoma cells. In addition to antiproliferative properties, the FAAH inhibitor CAY10401 decreased prostate cancer cell invasion (Endsley et al., 2008). URB597 and AA-5HT were further found to decrease human lung cancer cell invasion via CB_2_- and TRPV1-dependent upregulation of TIMP-1 (Winkler et al., 2016). An involvement of CB_1_ in the antiproliferative action on colorectal carcinoma cells was proven for AA-5HT (Ligresti et al., 2003).

In agreement with the proposed inhibitory impact of 2-AG on cancer progression, the MAGL inhibitor JZL184 was found to elicit antiproliferative and proapoptotic effects on colorectal cancer cells (Ye et al., 2011). Concerning effects on cancer cell invasion, JZL184 was also reported to exert inhibitory action on prostate carcinoma cells *in vitro* and *in vivo* (Nomura et al., 2011a). A contribution of the cannabinoid receptors in this context is under controversial discussion. On the one hand, a partial involvement of CB_1_ in the anti-invasive and growth-inhibitory impact of MAGL inhibition has been reported (Nomura et al., 2011a). On the other hand, the anticarcinogenic features of different cancer cells were shown to be rescued by add-back of free fatty acids *in vitro* and by a high-fat diet *in vivo* (Nomura et al., 2010). These findings are in line with a report, which presented MAGL activity as a source of arachidonic precursors that cause inflammation (Nomura et al., 2011b). In another study, JZL184 was found to inhibit proliferation of prostate cancer cells exclusively when these were activated with EGF (Cipriano et al., 2014). Other MAGL inhibitors that were proven to exert anticancer properties are the reversible MAGL inhibitor pristimerin, which elicited cancer cell apoptosis (Yousef et al., 2017), and URB602, which caused inhibition of tumor angiogenesis via downregulation of vascular endothelial growth factor (VEGF) and fibroblast growth factor (Pagano et al., 2017).

## Transport Proteins

As intracellular transporters that coordinate the delivery of AEA and 2-AG, as well as of OEA and PEA, to their catabolic enzymes, members of the fatty acid-binding protein (FABP) family have been the matter of debate in recent years. Furthermore, FABPs were discussed as proteins that shuttle between cytosol and nucleus and thereby deliver endocannabinoid compounds to their intracellular targets such as PPARs. In particular, FABP inhibitors have raised scientific interest as a treatment option for analgesic and anti-inflammatory purposes (Kaczocha et al., 2014).

Currently, 10 FABP subtypes with tissue-specific distributions are known (for review, see Thumser et al., 2014). For several FABPs, the binding capacities of endocannabinoids and endocannabinoid-like substances have been reported (Kaczocha et al., 2009, 2012; Sanson et al., 2014; Huang et al., 2016). In this context, a gender-specific endocannabinoid regulation was elucidated for FABP1. Accordingly, brains of male FABP1 knockout mice were shown to content higher concentrations of AEA, 2-AG, OEA, and PEA compared to wild-type mice (Martin et al., 2016b). In contrast to this, another study from the same group was able to demonstrate that AEA and 2-AG levels were unaltered and OEA and PEA were decreased in brains of female FABP1 knockout mice as compared to wild-type animals (Martin et al., 2016a). In addition, Δ^9^-THC and Δ^9^-THC-OH, as well as downstream metabolites of Δ^9^-THC such as Δ^9^-THC-COOH and Δ^9^-THC-CO-glucuronide were found as binding partners of hepatic FABP1 (Huang et al., 2018). Furthermore, a potentiated uptake of AEA was described for FABP5-overexpressing neuroblastoma cells (Kaczocha et al., 2009). In FABP5 and FABP7 knockout mice, upregulation of AEA, PEA and OEA have been linked to reduced nociception, with the antinociceptive action being sensitive to antagonists to CB_1_, PPARα and TRPV1 (Kaczocha et al., 2015). For the endocannabinoid-like substance OEA, one investigation supported the notion of FABP5 as an intracellular shuttling protein that mediates activation of PPARα (Kaczocha et al., 2012).

As reviewed previously (Schwarz et al., 2018), a number of FABPs are involved in carcinogenesis. Thus, FABP1 has been found to increase tumor angiogenesis in hepatocarcinoma by upregulation of VEGF (Ku et al., 2016), and FABP4 was shown to enhance cancer aggressiveness in different tumor entities such as myeloid leukemia (Yan et al., 2017), prostate (Herroon et al., 2013; Uehara et al., 2014) and breast cancer (Guaita-Esteruelas et al., 2017). FABP5 knockdown was shown to inhibit tumor cell proliferation and invasiveness of cervical cancer cells (Wang W. et al., 2016) and oral squamous cell carcinoma (Fang et al., 2010) as well as tumor cell proliferation of prostate cancer cells (Kawaguchi et al., 2016). In agreement with these findings, inhibition of FABP5 was associated with reduced carcinogenic potential of mammary carcinoma (Kannan-Thulasiraman et al., 2010; Levi et al., 2013; Zhang et al., 2015) and prostate cancer cells (Forootan et al., 2014). A recent investigation reported upregulation of VEGF in response to a FABP5-dependent activation of PPARγ as a crucial event in tumor neovascularization of prostate cancers (Forootan et al., 2016). Furthermore, increase of tumor cell migration was found to be associated with FABP7 overexpression (Mita et al., 2010).

The association of enhanced carcinogenesis and FABP overexpression, however, is not uniformly confirmed by other studies. Accordingly, binding of FABP3 to the integrin α-subunit was found to be associated with inhibition of breast cancer cell invasion (Nevo et al., 2010). FABP3 overexpression was furthermore reported to confer apoptosis in a teratocarcinoma cell line (Song et al., 2012). In addition, FABP7 was significantly upregulated, whereas FABP1 appeared downregulated in renal cell carcinoma as compared to normal tissue (Tölle et al., 2009). Downregulation of FABP1 was correlated with tumor differentiation and intratumoral inflammation (Inoue et al., 2014). Finally, the high expression of FABP1 in hepatocellular carcinoma was associated with a better prognosis than in patients with low FABP1 expression (Wang et al., 2014).

Although data concerning anticancer effects of FABP inhibitors acting via endocannabinoids are still missing, some investigations reported that FABP inhibitors actually developed for the treatment of obesity, atherosclerosis, diabetes, and metabolic syndrome (for review, see Wang Y.T et al., 2016) modulate the endocannabinoid system. Therefore, these FABP inhibitors could perhaps serve as potential tools to inhibit cancer progression. BMS309403, designed as an FABP4 inhibitor that also elicits its effects on HeLa cells that dominantly express FABP5 (Kaczocha et al., 2012), was shown to increase AEA in neuroblastoma cells (Kaczocha et al., 2009). Furthermore, SB-FI-26, an FABP5 inhibitor, was likewise found to increase AEA in rat sarcoma cells (Björklund et al., 2014).

## Clinical Implication and Outlook

Taking into account the different facts concerning cannabinoid action on cancer cells and tissue *in vitro* and *in vivo*, the endocannabinoid system encompasses several attractive pharmacotherapeutic targets for cancer treatment.

Currently, cannabinoids are used for palliative treatment of cancer patients. In this context, a recent meta-analysis included nine randomized controlled and crossover trials of palliative care using cannabinoid compounds and reported only low-quality evidence of a clinical benefit of cannabinoids in the treatment of cancer pain and overall no recommendations for the use of cannabinoids in palliative care treatment for cancer (Mücke et al., 2018). Another meta-analysis reported a reduction of at least 30% in chronic pain of diverse origins with cannabinoids compared to the placebo group. The authors stated moderate-quality evidence for cannabinoids (smoked Δ^9^-THC and nabiximols, an approximate 1:1 combination of Δ^9^-THC and CBD) as beneficial for the treatment of chronic neuropathic or cancer pain and low-quality evidence for the cannabinoids dronabinol and nabiximols as beneficial treatment options for chemotherapy-induced nausea and vomiting (Whiting et al., 2015). Thus, the efficacy of cannabinoid-based drugs in the treatment of these classical symptoms of cancer and cancer treatment has to be evaluated critically. However, besides reduction of chemotherapeutic-induced emesis, nausea and pain, cannabinoids may also reduce other severe side effects caused by currently used cytostatics. Accordingly, a recent study revealed cannabinoids as possible treatment options against cisplatin-induced traumatic cochlear insult (Ghosh et al., 2018). Cisplatin-induced nephrotoxicity is another adverse effect that may be counteracted by CBD (Pan et al., 2009) and the peripherally restricted cannabinoid CB_2_ receptor agonist LEI-101 (Mukhopadhyay et al., 2016) as has been proven in a rodent model. Furthermore, doxorubicin-induced cardiotoxicity has recently been shown to be suppressed by the cannabinoid WIN55,212-2 in mice (Rahmatollahi et al., 2016). Several studies further reported cannabinoids to reduce peripheral neuropathy in the context of HIV infections (Andreae et al., 2015) and diabetes (Wallace et al., 2015), thereby suggesting further use in the prevention of chemotherapeutic-induced neuropathy, as has been discussed recently (Abrams, 2016).

Concerning systemic effects of cannabinoids to combat disease severities of cancer, recently published orphan drug designations for cannabinoids as a treatment option for glioma pave the way for clinical evaluations of cannabinoids in cancer treatment. Accordingly, in the USA CBD (FDA, 2014) and a combination of Δ^9^-THC and CBD (FDA, 2015, 2018) have received orphan drug designations, while in Europe an orphan drug designation was granted only for the combination of Δ^9^-THC and CBD (EMA, 2016). One probable advantage for cancer patients in this context may lie in the broad array of anticancer effects of cannabinoids, which include inhibitory effects on cancer cell proliferation, angiogenesis, invasion and chemoresistance, while inducing apoptosis, autophagy and tumor immune surveillance. Particularly the latter effect has been discussed controversially in recent years. On the one hand, Δ^9^-THC was found to cause enhanced tumor growth in immunocompetent murine tumor models in terms of lung and breast cancer xenografts (Zhu et al., 2000; McKallip et al., 2005). On the other hand, using a melanoma xenograft model WIN55,212-2 exerted the opposite effects. Accordingly, the tumor-regressive action of WIN55,212-2 was more pronounced in immunocompetent than in immunodeficient mice (Blázquez et al., 2006). Another study further found reduction of breast cancer growth and metastasis in an immunocompetent mouse model in response to treatment with the CB_2_ receptor agonist JWH-015 (Hanlon et al., 2016). Concerning the mechanisms leading to enhancement of tumor-immune interactions, a recent study found an increase of tumor cell killing via lymphokine-activated killer cells crosslinked by LFA-1 to CBD-, Δ^9^-THC- and R(+)-methanandamide-induced ICAM-1 on the surface of lung cancer cells (Haustein et al., 2014). Finally, one study reported reduced infiltration of macrophages and neutrophils in cancer tissue of animals treated with Δ^9^-THC (Glodde et al., 2015).

However, the efficacy of these drugs will have to be thoroughly evaluated in clinical studies. Notably, a recent publication presented a collection of clinical cases of cancer patients who had received synthetic, pharmaceutical-grade CBD, with several cases exhibiting reduction in cancer size or in the number of circulating cancer cells (Kenyon et al., 2018). However, clinical studies with the respective control groups have to be conducted to define clearly the systemic anticancer effects, particularly with respect to efficient doses. So far the scientific literature provides merely a single pilot study that addressed the safety of intracranially administered Δ^9^-THC in patients suffering from glioblastoma multiforme. Due to the design and the size of the clinical trial that enrolled nine patients with recurrent glioblastoma multiforme, a conclusion concerning the effect of Δ^9^-THC on patients’ survival cannot be drawn (Guzman et al., 2006).

One important factor that further argues for the use of cannabinoid-based drugs in cancer therapy may lie in their synergistic action on the efficacy of currently used chemotherapeutic drugs. Particularly, Δ^9^-THC and CBD were found to synergistically act on anticarcinogenic properties of bortezomib (Morelli et al., 2014), carfilzomib (Nabissi et al., 2016), carmustine (Nabissi et al., 2013), cisplatin (Deng et al., 2017), cytarabine (Liu W. M. et al., 2008), doxorubicin (Liu W. M. et al., 2008; Nabissi et al., 2013; Elbaz et al., 2016), mitoxantrone (Holland et al., 2007), temozolomide (Torres et al., 2011; Nabissi et al., 2013; López-Valero et al., 2018), and vinca alkaloids (Holland et al., 2006; Liu W. M. et al., 2008).

Taken together, cannabinoids and compounds affecting the endocannabinoid system may complement the range of currently used chemotherapeutic agents as a pharmacotherapeutic option for cancer treatment with broadly diversified mechanisms.

## Author Contributions

RR wrote the manuscript in consultation with and under input of RS and BH. All authors discussed the manuscript, table and figure and commented on the manuscript. BH supervised the manuscript.

## Conflict of Interest Statement

The authors declare that the research was conducted in the absence of any commercial or financial relationships that could be construed as a potential conflict of interest.
